# Multiparametric MRI: practical approach and pictorial review of a useful tool in the evaluation of brain tumours and tumour-like lesions

**DOI:** 10.1186/s13244-020-00888-1

**Published:** 2020-07-17

**Authors:** Vijay Sawlani, Markand Dipankumar Patel, Nigel Davies, Robert Flintham, Roman Wesolowski, Ismail Ughratdar, Ute Pohl, Santhosh Nagaraju, Vladimir Petrik, Andrew Kay, Saiju Jacob, Paul Sanghera, Victoria Wykes, Colin Watts, Harish Poptani

**Affiliations:** 1 University Hospitals Birmingham NHS Foundation Trust, Queen Elizabeth Hospital Birmingham, Mindelsohn Way, Edgbaston, Birmingham, B15 2TH UK; 2 University of Birmingham, Edgbaston, Birmingham, B15 2TT UK; 3 Centre for Pre-Clinical Imaging, Department of Cellular and Molecular Physiology, University of Liverpool, Crown Street, Liverpool, L69 3BX UK

**Keywords:** Diffusion MRI, Perfusion MRI, MR Spectroscopy, Multiparametric MRI, Neuroimaging

## Abstract

MRI has a vital role in the assessment of intracranial lesions. Conventional MRI has limited specificity and multiparametric MRI using diffusion-weighted imaging, perfusion-weighted imaging and magnetic resonance spectroscopy allows more accurate assessment of the tissue microenvironment. The purpose of this educational pictorial review is to demonstrate the role of multiparametric MRI for diagnosis, treatment planning and for assessing treatment response, as well as providing a practical approach for performing and interpreting multiparametric MRI in the clinical setting. A variety of cases are presented to demonstrate how multiparametric MRI can help differentiate neoplastic from non-neoplastic lesions compared to conventional MRI alone.

## Key points

- Conventional MRI has a limited role in differentiating tumours from various non-tumoural lesions.
- Multiparametric MRI using diffusion-weighted imaging, perfusion-weighted imaging and magnetic resonance spectroscopy allows more accurate assessment of intracranial lesions.
- Apparent diffusion coefficient, relative cerebral blood volume and choline:creatine ratio are the main multiparametric MRI parameters which are useful for distinguishing between different entities.
- Multiparametric MRI is also helpful for grading and treatment response assessment of brain tumours, due to its ability to assess the tissue microenvironment.

## Introduction

MRI plays a major role in the diagnosis, grading, treatment and treatment response assessment of brain tumours and other intracranial lesions. Conventional MRI provides the anatomical and structural details of lesions in the neuraxis; however, its specificity is limited. Even with recent improvements in contrast resolution, higher magnetic field strengths and improved contrast agents, tissue characterisation remains limited using conventional imaging acquisitions. As a result of diagnostic uncertainties, patients will undergo invasive biopsy of brain lesions, which is not without risk [1]. Several adjunct MR imaging techniques have been developed to quantitatively measure a number of biophysical properties of brain tissue in vivo, allowing regional changes in the tissue microstructural environment to be better characterised. These techniques include diffusion-weighted imaging (DWI), perfusion-weighted imaging (PWI) and magnetic resonance spectroscopy (MRS). DWI provides information about cellularity and water movement, PWI provides information about angiogenesis and vascularity and MRS provides information about the composition of various metabolites within the tissue. These quantitative methods provide information about tumour cellularity, proliferation, vascularity, vessel permeability and cell membrane turnover. Changes in physiological processes due to the nature of the underlying lesion are reflected in the information obtained. There have been a number of studies demonstrating that these techniques in combination can help improve differentiation of neoplastic from non-neoplastic lesions (for example, tumefactive demyelination, tumefactive vasculitis and other inflammatory disorders) [2, 3], grading of brain tumours [4], differentiation of glioblastoma pseudoprogression from true progression [5] and response of brain metastases to stereotactic radiosurgery (SRS) treatment [6]. Over time, there has been development of these adjunct advanced MRI techniques in isolation, beginning with MRS, DWI and then PWI. In clinical practice and throughout the literature, usually these techniques were compared with each other; however, recent studies show that the information gained from each of these techniques are complementary. In this pictorial review, we illustrate the use of a multiparametric MRI approach consisting of DWI, PWI and MRS in clinical neuro-oncology practice to help with the diagnosis of intracranial lesions, treatment planning and assessing response to treatment.

## MRI protocol

**Fig. 1:**
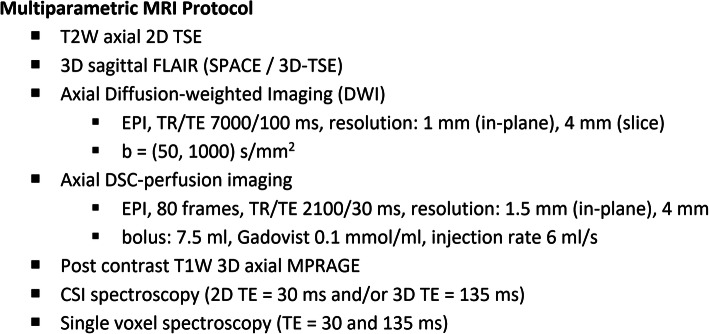
Multiparametric MRI protocol for intracranial lesions

Our multiparametric studies are performed on a 3 T scanner (Magnetom Verio; Siemens, Erlangen, Germany) with a 32-channel phased-array head coil, although such studies can also be performed on other similar scanners and coils. Acquisition parameters are summarised in Fig. 1. Axial T2-weighted (T2W) images, T2W FLAIR and DWI (*b* value 1000) of the whole brain are generally obtained first. This is followed by dynamic susceptibility contrast-enhanced (DSC) perfusion imaging using gradient-echo echo-planar imaging (GE-EPI) during the first pass of a standard dose (7.5 mmol) bolus of gadolinium-based contrast agent (Gadovist, Bayer Schering Pharma, Berlin, Germany) administered intravenously at a flow rate of 6 ml/s. A total of 80 imaging volumes are acquired at a temporal resolution of 2.1 s with the bolus typically arriving between the 10th and 15th volume. This is followed by post-contrast 3D T1-weighted (T1W) magnetisation-prepared rapid acquisition with gradient echo (MPRAGE) sequence acquired in the axial plane with sagittal and coronal reformats.

MRS is performed using a combination of multi-voxel (for tumoural and peri-tumoural regions) and single-voxel point resolved spectroscopy PRESS sequences with short echo (TE = 30 ms) and intermediate echo (TE = 135 ms). TE 135 ms is usually performed to show lactate inversion at 1.3 ppm (J-coupling effect). Typically, 2D or 3D MR spectroscopic imaging (MRSI) is first performed in the axial plane choosing a slice or slab with the largest contrast-enhancing lesion area (or FLAIR if non-enhancing), area with restricted diffusion, or high perfusion. This is followed by single-voxel MRS with placement of the volume-of-interest further guided by the metabolic profiles estimated by MRSI. The single voxel method is used to maximise diagnostic yield by combining information from contrast-enhancement, DWI, DSC and MRSI to sample the most relevant part of the lesion likely to provide the highest quality spectra.

## MRI post-processing and analysis

Apparent diffusion coefficient (ADC) maps are calculated from the DWI on the MR scanner software (Magnetom VB17; Siemens, Erlangen, Germany). DSC data are post-processed on a Siemens Leonardo workstation (software version VB17; Siemens, Erlangen, Germany) using a global arterial input function (AIF) without leakage correction, producing maps of relative cerebral blood volume (rCBV) and relative cerebral blood flow (rCBF). MRS data are processed and fitted using the MR scanner software (Magnetom VB17; Siemens, Erlangen, Germany) to include peak integral values for *N*-acetylaspartate (NAA), creatine (Cr), choline (Cho), myo-inositol (mI) + glycine (Gly), glutamine + glutamate (Glx) and lipids. ADC and rCBV values are measured using a 3 mm region-of-interest (ROI). MRS data are used to determine the maximum observed ratio of Cho/Cr.

## Clinical interpretation

Normal brain ADC values for cortical grey and white matter are 833 × 10^−6^ mm^2^ s^−1^ and 701 × 10^−6^ mm^2^ s^−1^ respectively [7]. Mean ADC values in high-grade neoplastic lesions such as glioblastoma, anaplastic astrocytoma, and metastases have shown to be 700–780 × 10^−6^ mm^2^ s^−1^, lymphoma has shown to be 510 × 10^−6^ mm^2^ s^−1^ and low-grade tumours have shown to be 1090 × 10^−6^ mm^2^ s^−1^ [8]. To calculate the rCBV ratio, the ROI is generally compared with the normal-appearing contralateral white matter. The mean rCBV ratios in high-grade neoplastic lesions have shown to be 1.9, compared to 1.3 in low-grade neoplastic lesions [9]. Normative values for Cho/Cr at TE 135 ms range from 0.7–1.0 in grey matter and 1.2–1.4 in white matter, with slightly higher values seen in the brainstem and cerebellum [10]. Short TE (30 ms) shows more metabolites and is primarily used for assessing tumoural and non-tumoural lesions. Normal Cho/Cr ratios using short TE MRS are 0.6 in grey matter and 1.0 in white matter [11]. High-grade neoplastic lesions have shown to demonstrate a mean Cho/Cr ratio of 2.4 on short TE MRS, compared with a mean Cho/Cr ratio of 1.5 for low-grade neoplastic lesions [12]. As there is a wide variability of cut-off values for each parameter in the literature, based on the results of a number of studies, we defined high-grade neoplastic lesions to have cut-off values of ADC < 1000 × 10^−6^ mm^2^ s^−1^, rCBV ratio > 2.0 and Cho/Cr ratio > 1.8 [12–15]. We utilised these parameters semi-quantitatively by defining the lowest ADC, highest rCBV and highest choline values within the lesion. This multiparametric information was read in combination with conventional imaging, clinical findings and other investigations.

## Neoplastic lesions

### Lymphoma

**Fig. 2:**
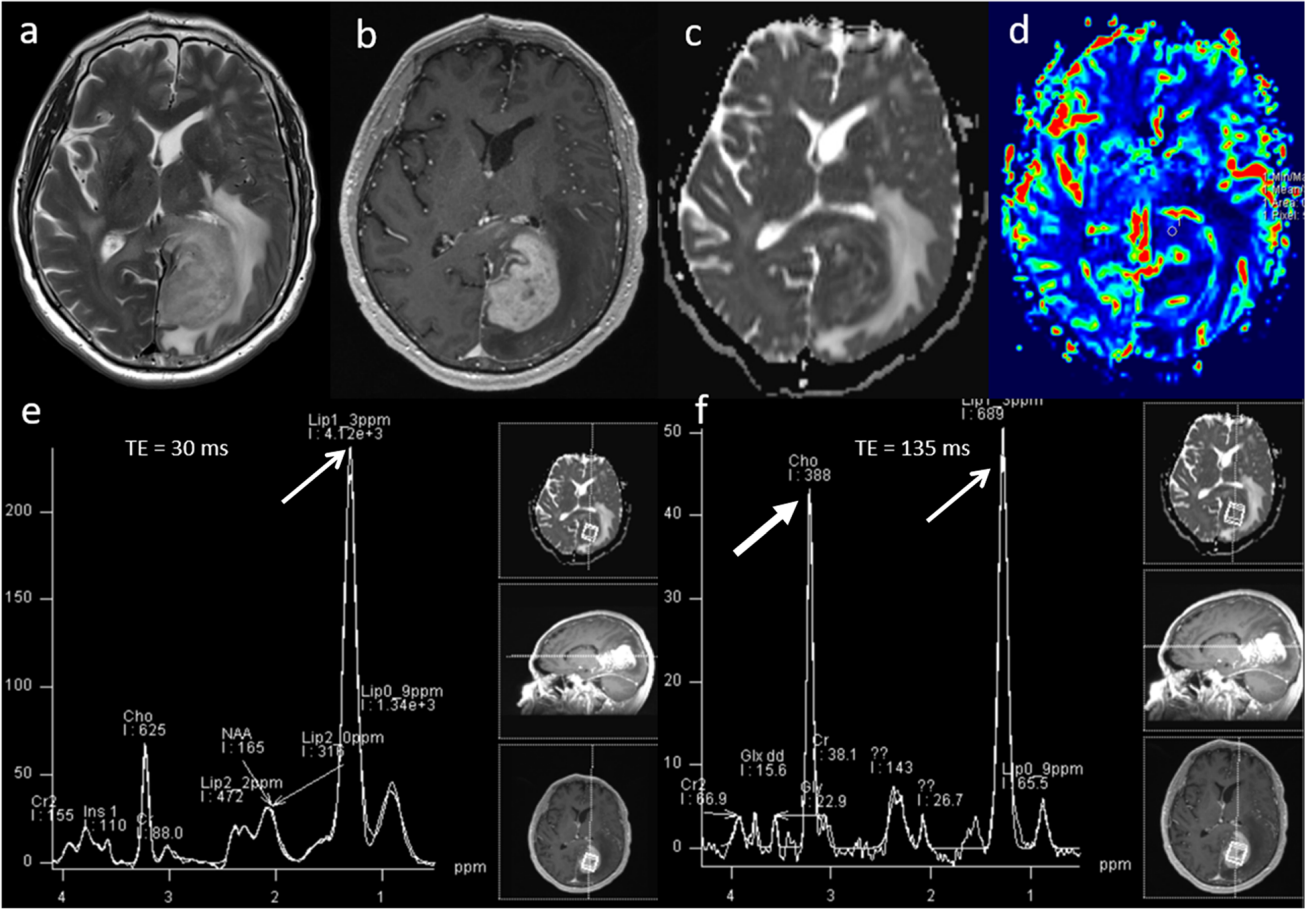
Lymphoma. Conventional MRI Findings: **a**, **b** Axial T2W and post-contrast T1W sequences show a large homogenously enhancing lesion in the left occipital lobe. **c** ADC map shows very low ADC (< 600 × 10^−6^ mm^2^ s^−1^) throughout the lesion. **d** PWI shows low perfusion throughout the lesion compared to normal-appearing contralateral white matter. **e**, **f** MRS shows very high Cho/Cr ratio (> 6, thick arrow) and very high lipid peaks in a non-necrotic appearing lesion (TE 30 ms and 135 ms, thin arrows). The low perfusion, very low ADC, very high lipid peak in a non-necrotic appearing lesion and high choline peak are characteristic of lymphoma. Histopathology confirmed a diffuse large B cell PCNSL

Primary central nervous system lymphoma (PCNSL) is a form of extranodal non-Hodgkin’s lymphoma and unlike other brain neoplasms, resection of PCNSL rarely provides benefit, instead chemotherapy and radiotherapy are preferred treatment choices [16]. Hence, it is important to differentiate lymphoma from high-grade glioma. Conventional imaging appearances for PCNSL are an avidly homogenously enhancing mass, which is T1 hypointense and T2 iso- to hypointense. There is little mass effect for size and limited surrounding vasogenic oedema. Multiparametric MRI in PCNSL demonstrates a very low ADC suggesting dense cellular packing, lower perfusion due to lack of angiogenesis, very high Cho/Cr ratio due to high membrane turnover, high lipid peak at 1.3 ppm due to infiltration by macrophages even without necrosis [17] and very low NAA levels [18]. Imaging features of typical PCNSL is demonstrated in Fig. 2. However, it is important to note that PCNSL in immunocompromised patients may be more heterogeneous, with central necrosis and haemorrhage.

### Low-grade glioma

**Fig. 3:**
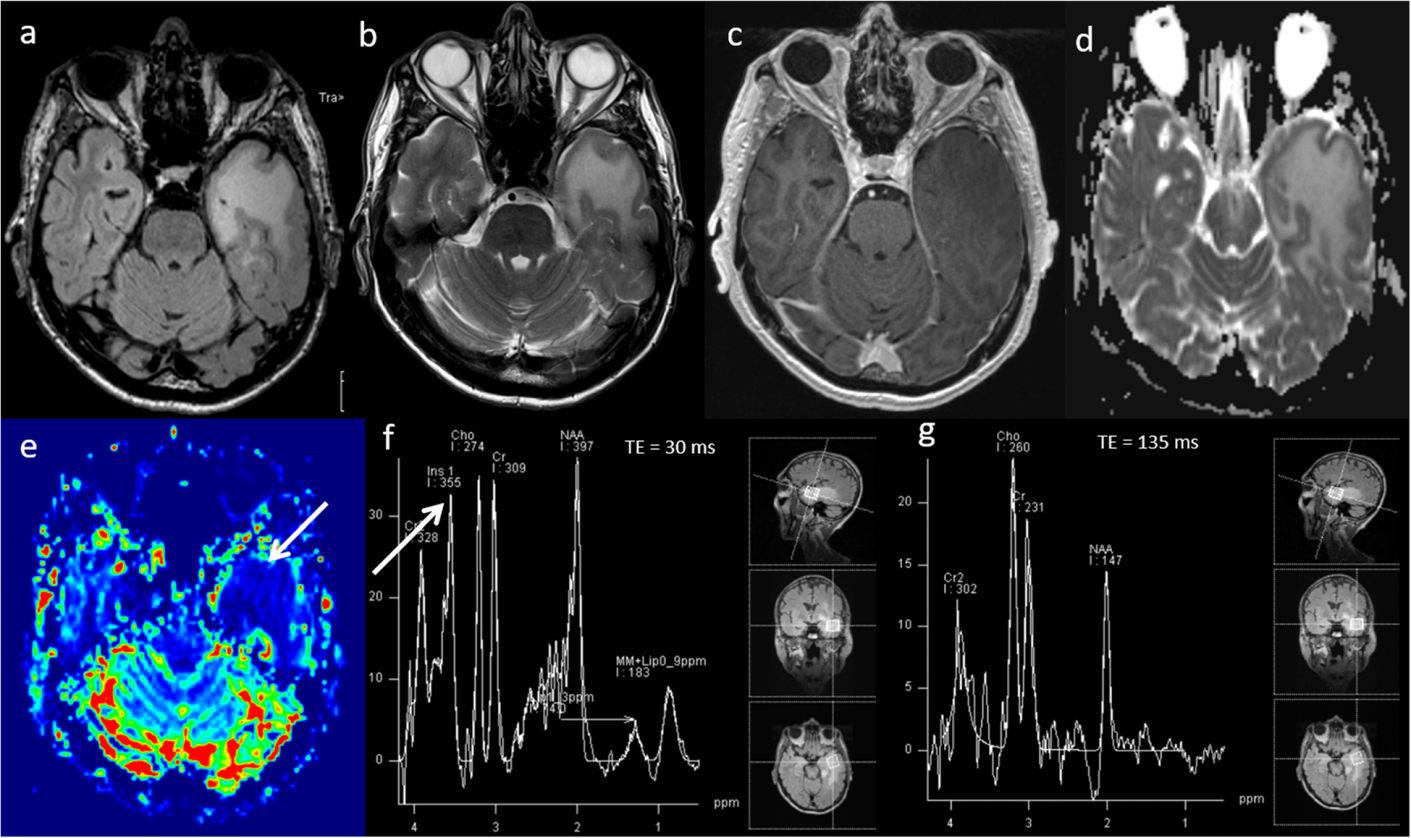
Low-grade glioma. Conventional MRI: (**a**) FLAIR, (**b**) T2W and (**c**) post-contrast T1W sequences show a diffuse abnormality in the left temporal lobe without contrast enhancement. **d** ADC map shows high ADC throughout the lesion (1300 × 10^−6^mm^2^s^−1^). **e** PWI shows low perfusion throughout the lesion (arrow) compared to normal-appearing white matter, and (**f**, **g**) MRS (TE 30 ms) shows slightly raised Cho/Cr ratio (1.0), slightly low NAA/Cr (1.1) and very high mI/Cr ratio (0.9, arrow). Lipid or lactate peaks are not significantly elevated. Multiparametric MRI appearances suggest no evidence of dedifferentiation. Stable appearances have been seen on follow-up imaging for over five years, confirming the lesion’s low-grade nature

Low-grade gliomas are primary neoplasms of the brain which are generally slow-growing and are typically diagnosed in young adults between ages 20 and 45 [19, 20], but most will transform to a high-grade lesion, with the median time being 56 months for grade II gliomas [21]. Low-grade gliomas are usually detected incidentally and appear as an area of focal signal abnormality with no enhancement on conventional MRI. Multiparametric MRI features of a low-grade glioma are a relatively high ADC (> 1000 × 10^−6^ mm^2^ s^−1^) [14], low rCBV (< 2) [14], low Cho/Cr ratio (< 1.8), high NAA and absence of lactate and lipids on MRS [22]. Imaging features of typical low-grade glioma is demonstrated in Fig. 3.

### Malignant transformation of low-grade glioma

**Fig. 4:**
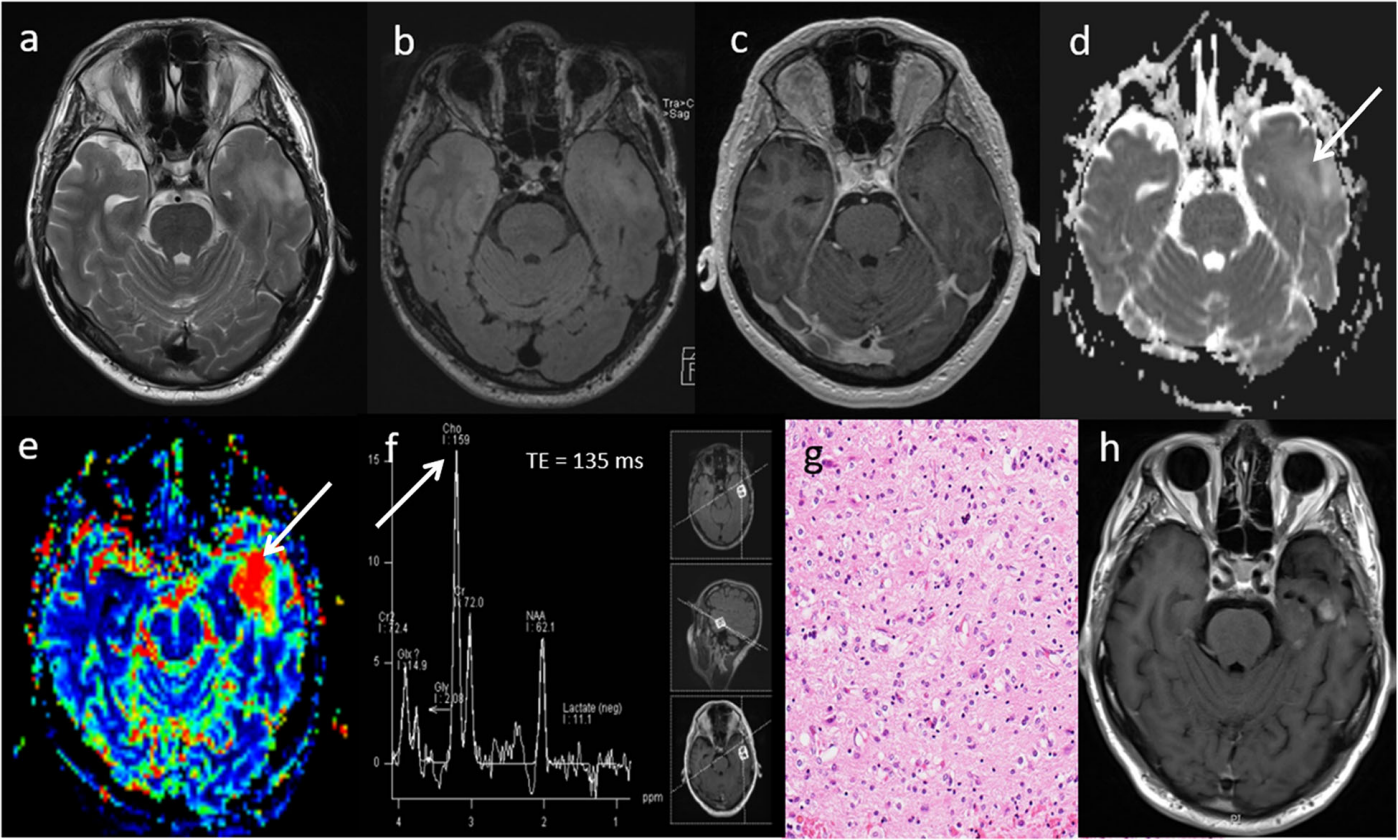
Malignant transformation of low-grade glioma. **a**–**c** T2W, FLAIR and post-contrast T1W sequences demonstrate non-enhancing signal abnormality in the left temporal lobe. Multiparametric MRI: **d** Heterogeneous ADC values throughout the lesion with focal areas of low ADC (lowest observed 940 × 10^−6^ mm^2^ s^−1^, arrow). **e** High rCBV throughout the lesion (arrow) compared to normal-appearing white matter (3.5). **f** Single-voxel spectroscopy shows very high Cho/Cr (2.3, arrow) and Cho/NAA ratios (3.1). **g** Histopathology from biopsy of the lesion shows low-grade diffuse astrocytoma with mild to moderately pleomorphic astrocytic cells in a fibrillary background. There was discrepancy of histological and genetic classification with morphological features of a low-grade glial neoplasm, but a convincing genetic profile of glioblastoma, overriding the morphological appearances. **h** Follow-up imaging 6 months later shows contrast enhancement indicating malignant transformation on conventional MRI

The presence of contrast enhancement in a brain tumour is often regarded as a sign of malignancy; however, non-enhancing gliomas are malignant in approximately one third of cases [23]. This has an impact upon treatment, patient outcome and overall survival, as conventional MRI has limitations for the grading of brain tumours. Transforming low-grade gliomas can show changes in multiparametric features before contrast enhancement is seen on conventional imaging. In the case of perfusion imaging, a significant increase in rCBV can be seen up to 12 months before transformation is seen on conventional imaging [24]. Multiparametric MRI features of a transforming low-grade glioma are focal low ADC (< 1000 × 10^−6^ mm^2^ s^−1^) [14], high rCBV (> 2) [14], high Cho/Cr ratio (> 1.8), low NAA and presence of lactate and lipids on MRS [22, 25]. In the early stages of malignant transformation, only one or two of the above parameters may be abnormal focally within the tumour, and any longitudinal changes in multiparametric information can suggest a transforming tumour. Early detection of malignant transformation, before contrast enhancement is seen on conventional MRI, will allow early initiation of appropriate treatment, which will ultimately have an effect on improving the patient’s overall survival. Typical multiparametric MRI features of a transforming low-grade glioma is demonstrated in Fig. 4.

### Targeting biopsy for a non-enhancing tumour

**Fig. 5:**
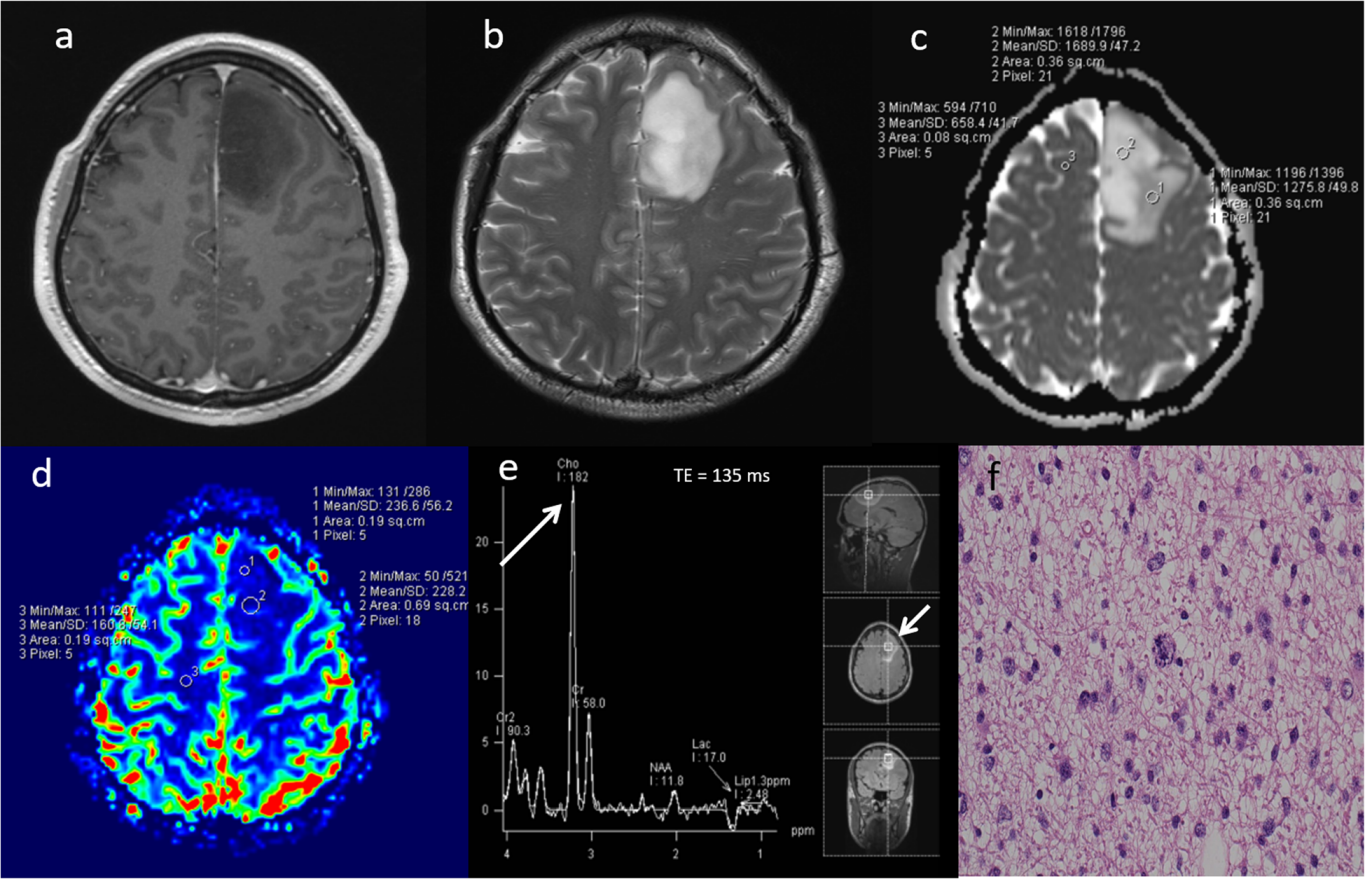
Targeting biopsy for a non-enhancing tumour. Conventional MRI: **a**, **b** Post-contrast T1W and T2W sequences demonstrate a large non-enhancing space occupying mass lesion without significant oedema. Multiparametric MRI: **c**, **d** Heterogeneous ADC and rCBV values throughout the lesion. **e** Multi-voxel MRS clearly shows focal area of very high Cho/Cr (3.1) and very small lactate peak. **f** Targeted biopsy taken from the area of highest choline peak (arrow). Histopathology shows anaplastic astrocytoma with moderately atypical astrocytic cells in a fibrillary background with a few abnormal mitoses (WHO grade III).

There is substantial risk of inaccuracy in stereotactic biopsy, with under-grading of WHO grade III tumours reported in 28% of cases [26]. The successful stereotactic biopsy diagnosis rate utilising multiparametric MRI techniques has shown to be more than 93% [27]. To get a better biopsy yield and to avoid sampling error for non-enhancing tumours, the target of biopsy can be selected from a high choline, high rCBV or low ADC location. A case demonstrating the use of choline map produced by multi-voxel spectroscopy for choosing the highest choline peak to target biopsy in a non-enhancing tumour is shown in Fig. 5.

### High-grade glioma

Rim-enhancing lesions have a wide differential diagnosis on conventional MRI, with various treatment strategies. The differential diagnoses of thick rim and nodular enhancing lesion include a chronic infective lesion, a granulomatous lesion, metastasis and primary high grade glioma. High-grade glioma is an aggressive neoplasm which requires early diagnosis and neurosurgical intervention.

**Fig. 6:**
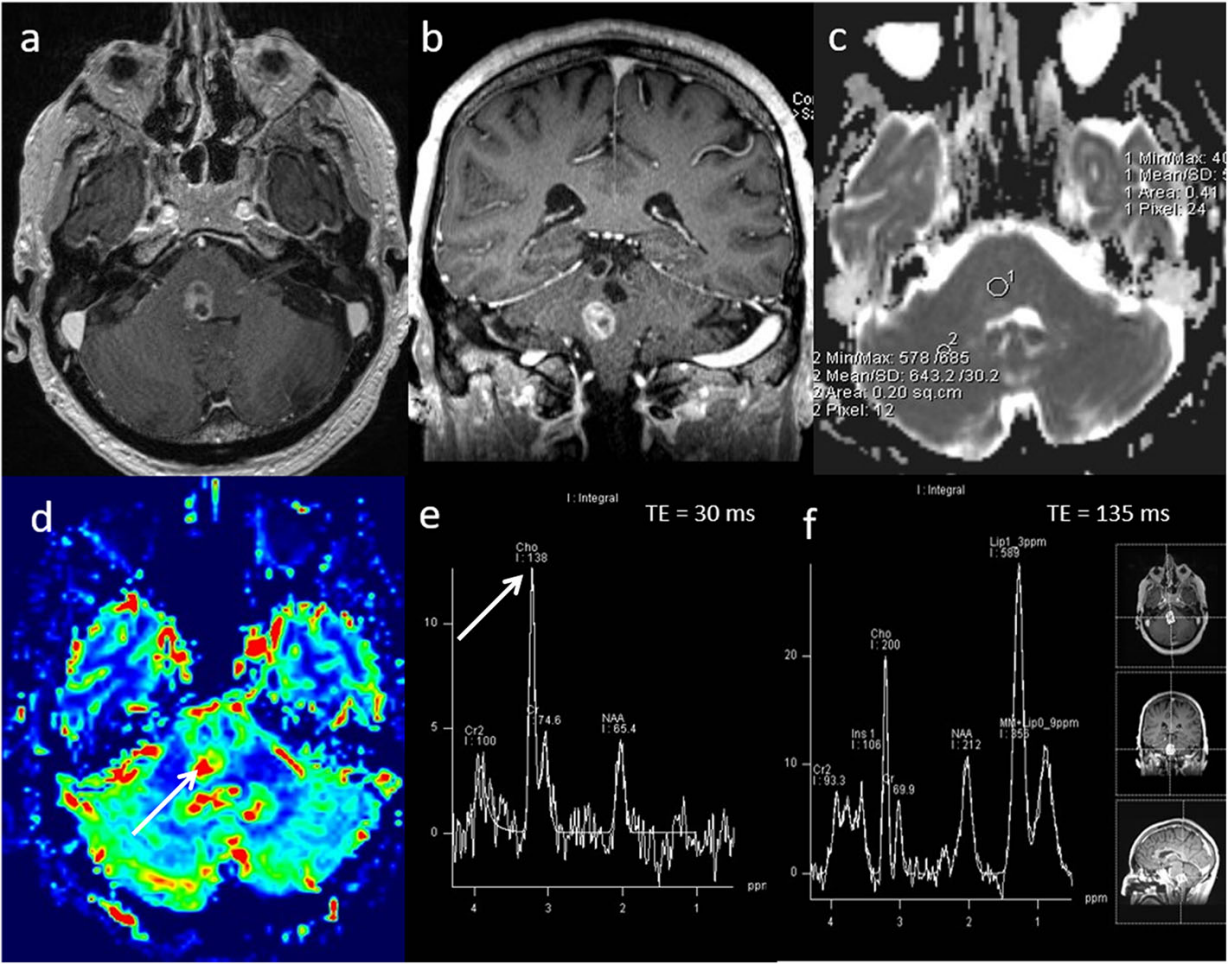
High-grade glioma. Conventional MRI: **a**, **b** Axial and coronal post-contrast T1W sequences, showing a well-defined lesion at the ponto-medullary junction. Multiparametric MRI: **c** ADC map demonstrates low ADC (590 × 10^−6^ mm^2^ s^−1^). **d** PWI shows high perfusion (rCBV 2.8, arrow). **e**, **f** MRS shows a high Cho/Cr ratio (2.9, arrow), low NAA/Cr ratio and presence of lipid peaks. MRI findings of a low ADC (< 1000 × 10^−6^ mm^2^ s^−1^), high rCBV (> 2) and high Cho/Cr ratio (> 1.8) are consistent with a high-grade glioma rather than a granuloma or abscess. The presence of high choline levels in the perilesional area (not shown) favour high-grade glioma over a metastatic lesion. In this patient, an initial biopsy was inconclusive and as a result of the multiparametric MRI findings, a decision to undergo further biopsy was overturned. The patient underwent radiotherapy for presumed glioblastoma

Typical multiparametric MRI appearances of a high-grade glioma are demonstrated in Fig. 6, which given the difficult location for biopsy had significant implications for changing the course of patient management.

### Gliomatosis cerebri

**Fig. 7:**
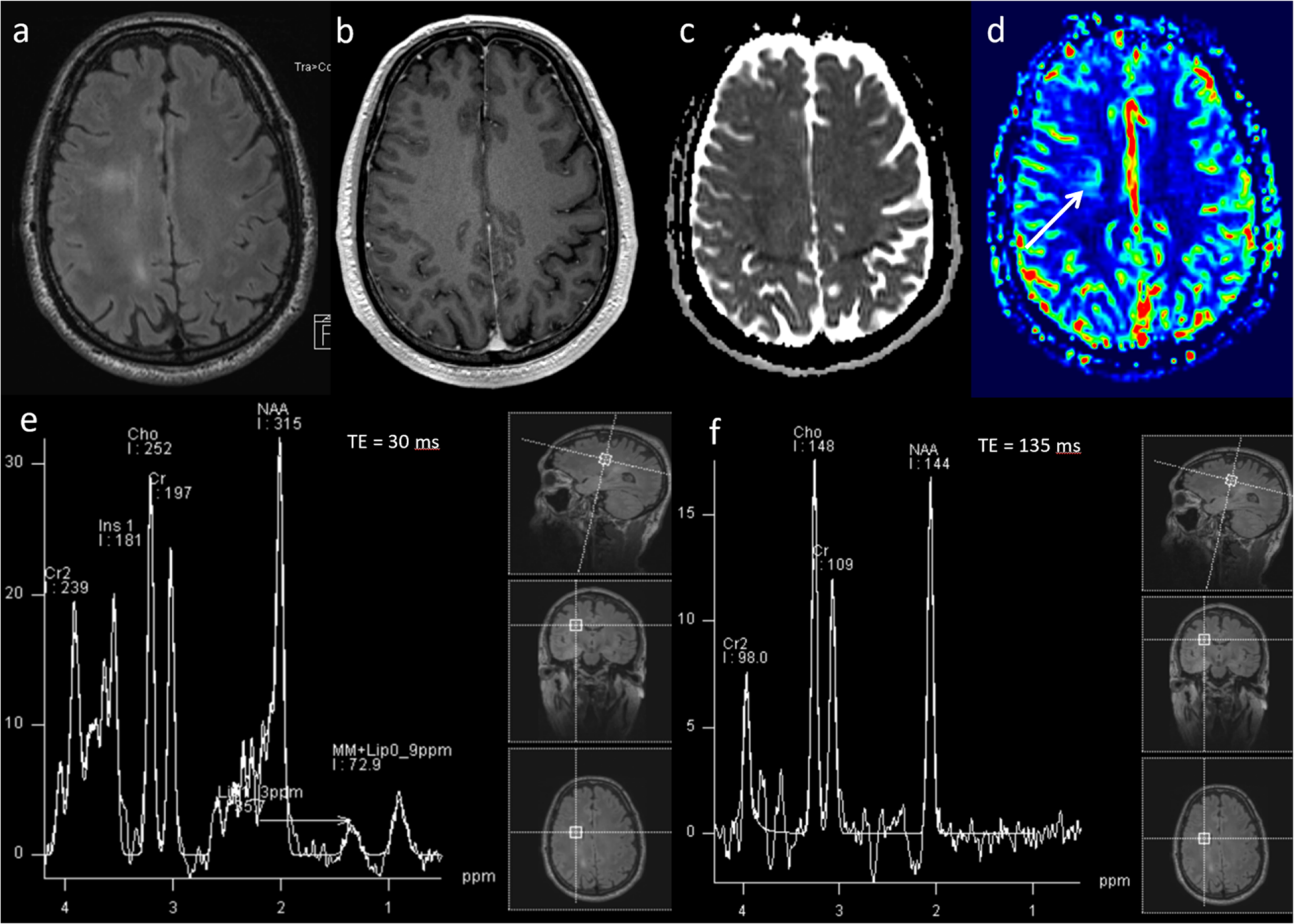
Gliomatosis cerebri. Conventional MRI: **a**, **b** Axial FLAIR and post-contrast T1W sequences, showing diffuse infiltrative non-enhancing deep white matter lesion. Multiparametric MRI: **c** ADC map demonstrates no areas of low ADC. **d** However, PWI shows a focal area of slightly raised perfusion in the right frontal centrum semiovale (arrow) compared to normal-appearing white matter. **e**, **f** MRS shows high mI/Cr ratio, slightly raised Cho/Cr ratio (1.2) and slightly low NAA/Cr ratio. Focal raised perfusion and choline area was chosen for the optimal site of biopsy

Gliomatosis cerebri is a rare growth pattern of infiltrative diffuse glioma with an incidence of 0.1 per million [28], containing areas of WHO grade II, III tumours and rarely grade IV tumours. It has relatively non-specific findings on conventional MRI and sometimes difficult to appreciate on histopathology unless used in combination with radiological findings. Multiparametric MRI can help in making the tumour diagnosis, identifying areas of early transformation and a suitable biopsy target, given the widespread changes [29]. A case of gliomatosis cerebri is shown in Fig. 7.

### Glioblastoma—treatment response

**Fig. 8:**
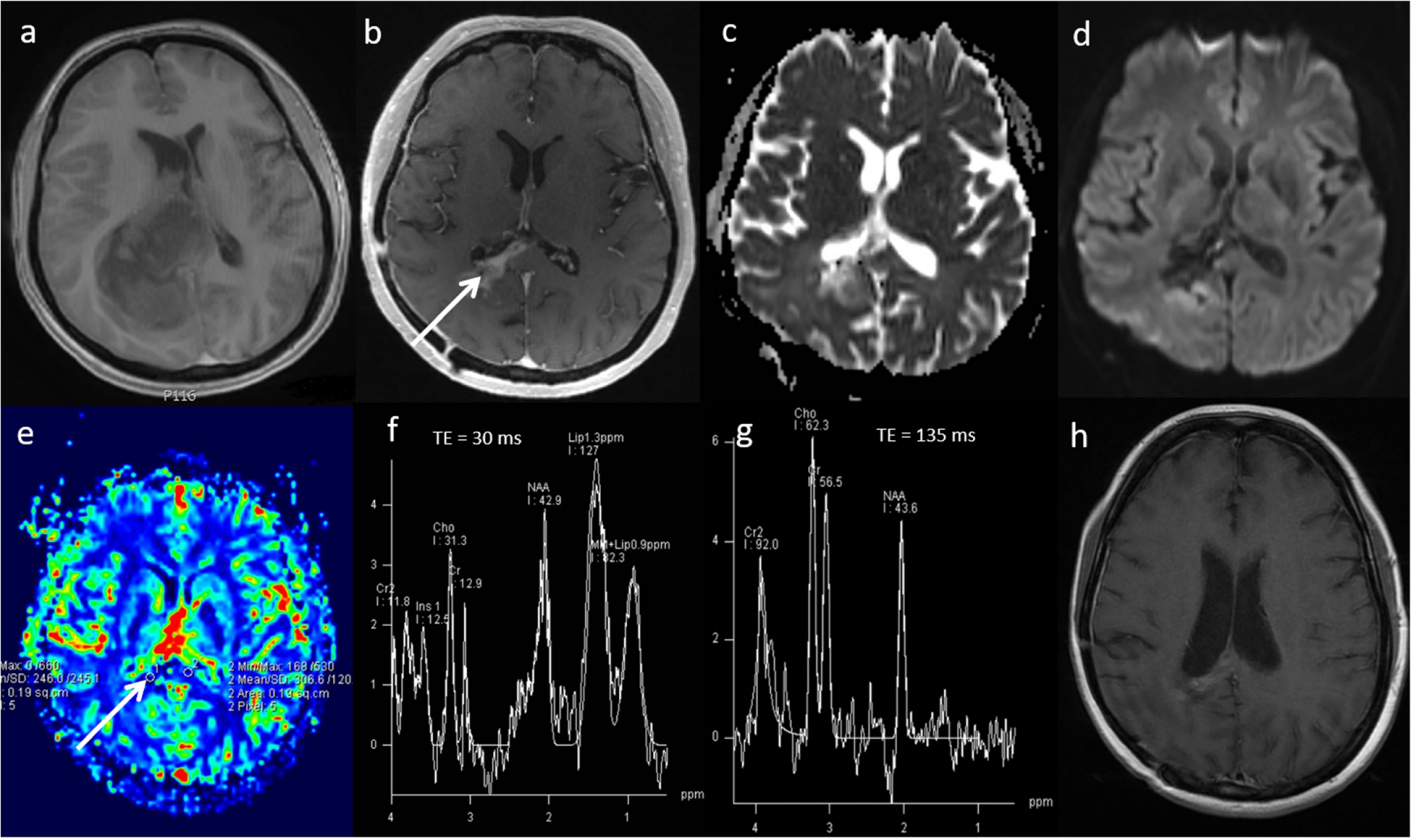
Glioblastoma—pseudoprogression. **a** Pre-operative T1-weighted image shows a right deep parietal region glioblastoma. **b** Conventional post-contrast T1-weighted image approximately 4 weeks after chemoradiotherapy treatment demonstrates a significant increase in the contrast-enhancing area (arrow). Multiparametric MRI at this timepoint demonstrates: **c**, **d** areas of high ADC (1186 × 10^−6^ mm^2^ s^−1^), (**e**) a low rCBV ratio (1.4, arrow) on PWI, (**f**, **g**) a low Cho/Cr ratio (1.4), a low Cho/NAA ratio and presence of lipid and lactate on MRS. Combination of parameters suggest pseudoprogression. **h** Clinical follow-up and conventional post-contrast T1W sequence at six months confirms a decrease in the amount of enhancing disease, indicating pseudoprogression

**Fig. 9:**
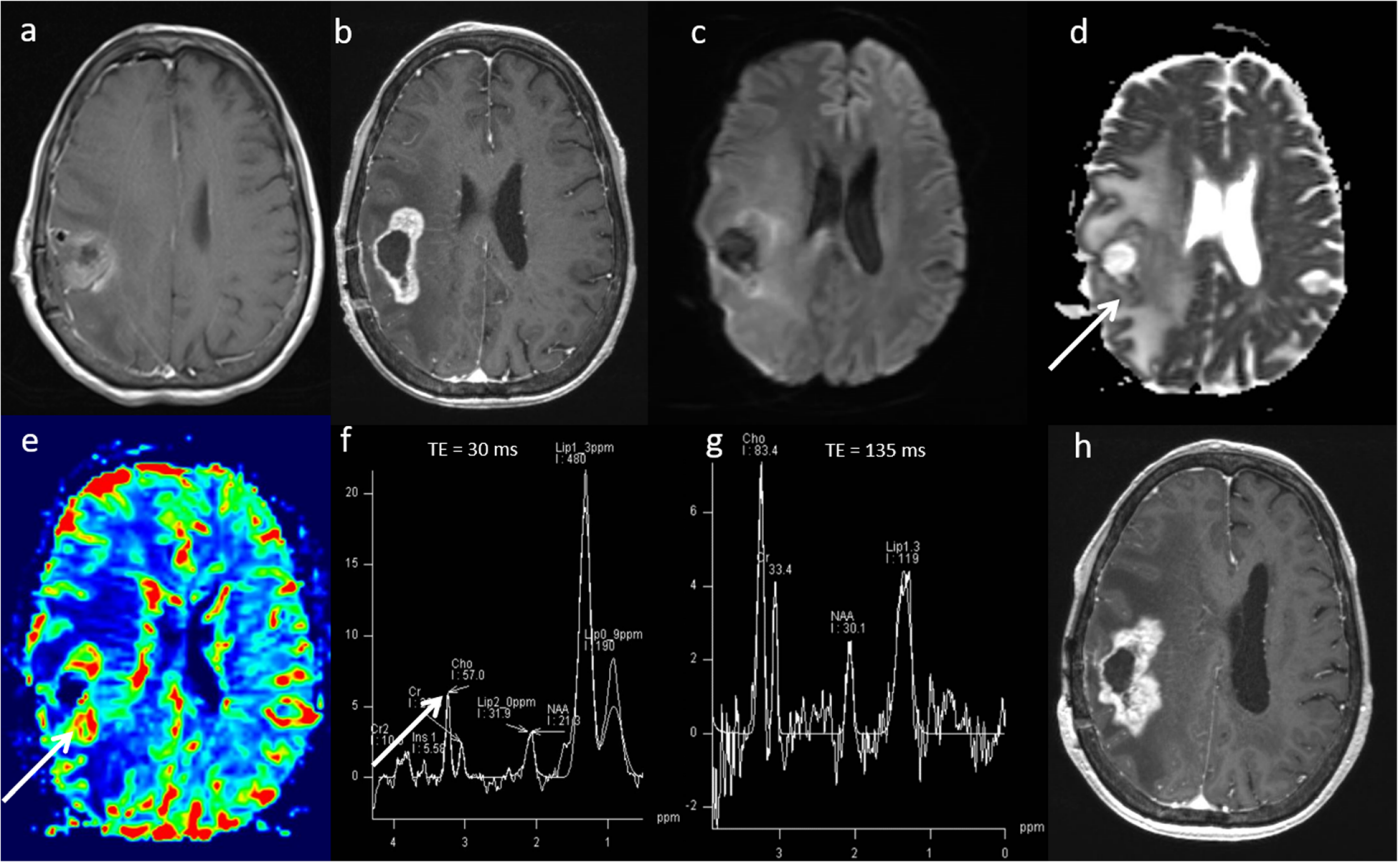
Glioblastoma—true progression. **a** Immediate post-operative contrast-enhanced T1-weighted scan following resection of a right fronto-parietal glioblastoma. **b** Conventional post-contrast T1-weighted image one month after chemoradiotherapy treatment demonstrates increase in the enhancing lesion size with associated oedema. Multiparametric MRI at this timepoint demonstrates: **c**, **d** areas of low ADC (903 × 10^−6^ mm^2^ s^−1^, arrow), (**e**) a high rCBV ratio (3.0, arrow) on PWI, (**f**, **g**) a high Cho/Cr ratio (2.3, arrow), high Cho/NAA ratio and presence of lipid/lactate on MRS. All parameters suggest a poor response and disease progression. **h** Six-month follow-up conventional post-contrast T1W sequence confirms an increase in enhancing disease, indicating true progression

Results from a recent meta-analysis show that following chemo-radiotherapy treatment of glioblastoma, 36% demonstrate pseudoprogression [30]. This is defined as an increase in enhancement on the first scan after treatment that subsequently resolves on its own without further treatment. Conventional MRI cannot differentiate between pseudoprogression and true tumour progression. Multiparametric MRI techniques probing the physiological and metabolic characteristics provide a more accurate assessment of changes following treatment than conventional MRI alone [31–37]. The typical multiparametric MRI appearances in pseudoprogression are high ADC (> 1000 × 10^−6^ mm^2^ s^−1^), low rCBV ratio (< 2) and a low Cho/Cr ratio (< 1.8) as demonstrated in the case shown in Fig. 8. On the contrary, typical multiparametric MRI appearances in true progression are general/focal low ADC (< 1000 × 10^−6^ mm^2^ s^−1^), high rCBV ratio (> 2) and a high Cho/Cr ratio (> 1.8) as shown in the case shown in Fig. 9.

### Metastasis—treatment response

**Fig. 10:**
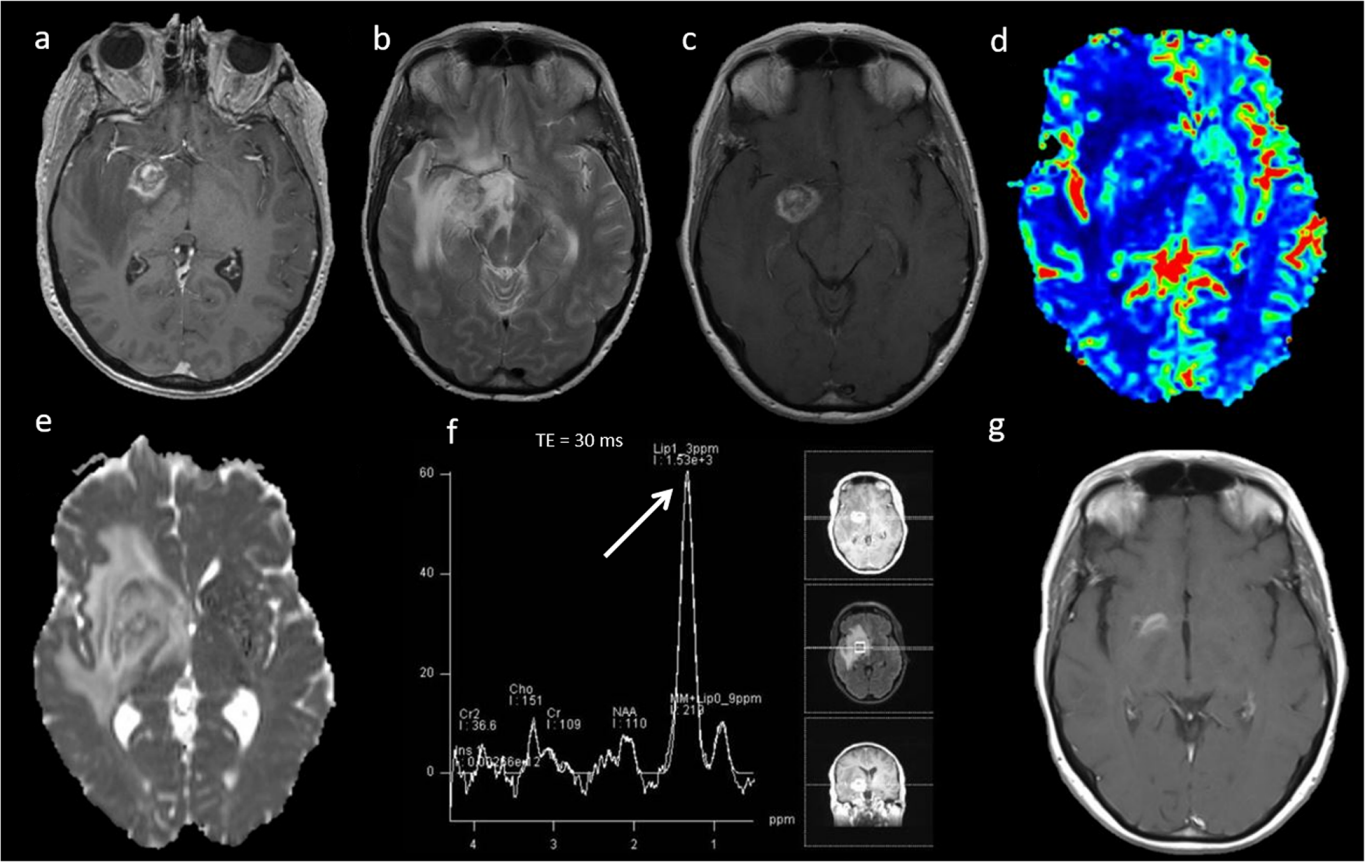
Brain metastasis—treatment effect. Melanoma metastasis. **a** Enhancing lesion in the right basal ganglion on axial T1 post-contrast MRI. **b**, **c** Post-SRS T2W and post-contrast T1W images shows increase in lesion size and oedema. Multiparametric MRI demonstrates: (**d**) an intermediate rCBV ratio (< 2.1) on PWI, (**e**) a high ADC (1159 × 10^−6^ mm^2^ s^−1^) and (**f**) low Cho/Cr ratio (1.6) and very high lipids (arrow) suggesting necrosis. **g** Three-month follow-up post-contrast T1W scan showed regression of the lesion

**Fig. 11:**
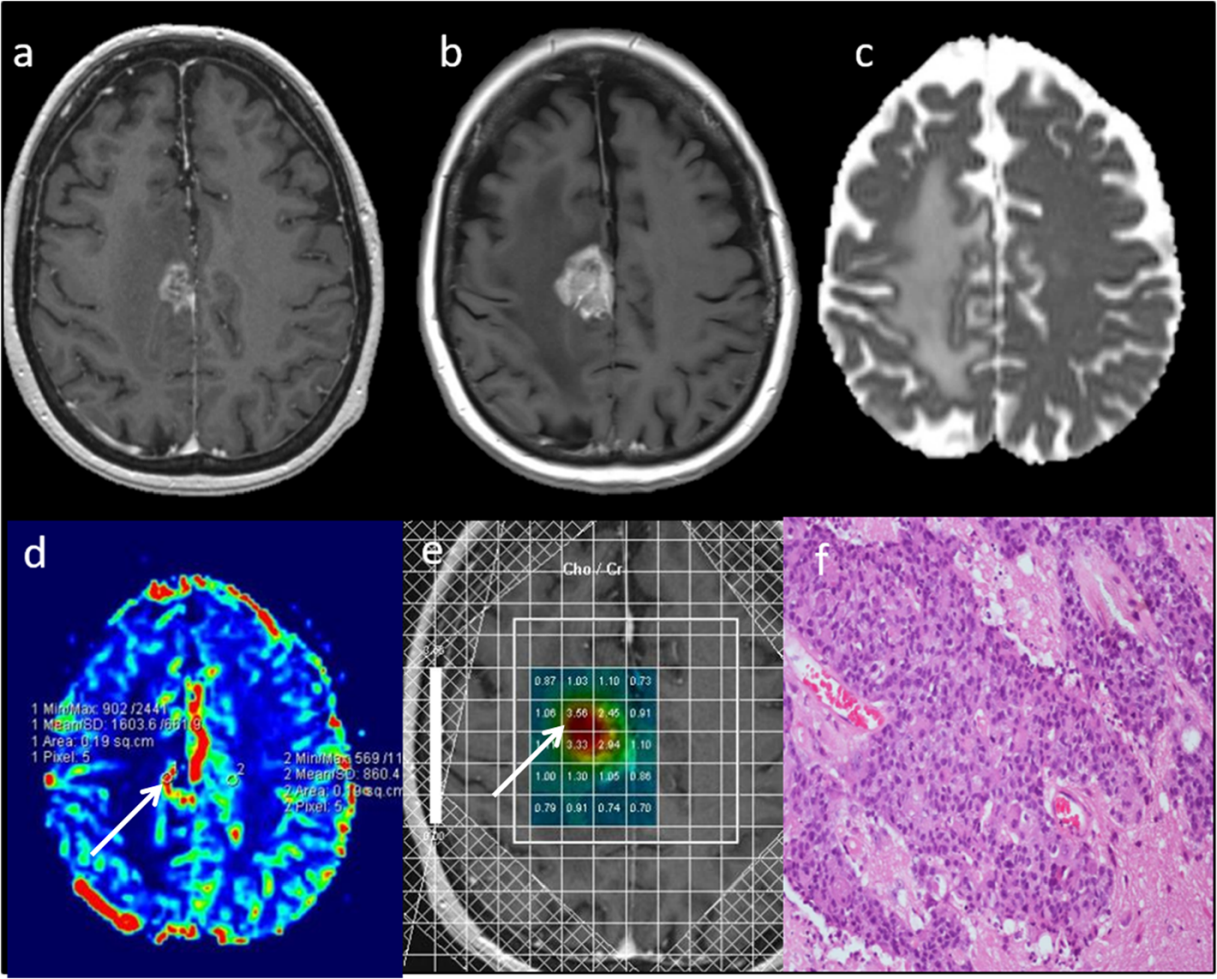
Brain metastasis—recurrent tumour. Brain metastasis from primary breast carcinoma. **a** Brain metastasis in the right mesial frontal lobe motor area on axial T1-weighted post-contrast MRI. **b** Post-SRS scan at 29 weeks demonstrates increase in the lesion size with oedema. Multiparametric MRI demonstrates: (**c**) a borderline ADC (999 × 10^−6^ mm^2^/s), (**d**) a borderline rCBV ratio (1.9, arrow) on PWI and (**e**) highest Cho/Cr ratio of 3.6 (arrow) on multi-voxel MRS (TE = 30 ms). Two of the three parameters (DWI and MRS) suggest poor response and disease progression. **f** Surgical decision was taken to operate on the motor cortex and excision of right frontal tumour was performed. Histopathology demonstrates poorly differentiated metastatic adenocarcinoma with discernible focal ductal structures and tumour well demarcated from adjacent brain tissue

Stereotactic radiosurgery (SRS) has become increasingly important in the management of brain metastases [38]. Following SRS, one-third of brain metastases increase in size, suggesting treatment failure [39]. Conventional MRI cannot differentiate between SRS-induced changes and tumour recurrence; however, combining multiparametric MRI techniques has shown promise in answering this clinical question [6]. The typical appearances in SRS-related treatment effect are high ADC (> 1000 × 10^−6^ mm^2^s^−1^), low rCBV ratio (< 2.1) and a low Cho/Cr ratio (< 1.8) and presence of lipid suggesting necrosis as demonstrated in the case shown in Fig. 10. On the contrary, typical multiparametric MRI appearances in recurrent tumour are general/focal low ADC (< 1000 × 10^−6^ mm^2^ s^−1^), high rCBV ratio (> 2.1) and a high Cho/Cr ratio (> 1.8) suggesting cellularity and membrane turnover as shown in the case shown in Fig. 11.

## Non-neoplastic lesions

### Abscess

**Fig. 12:**
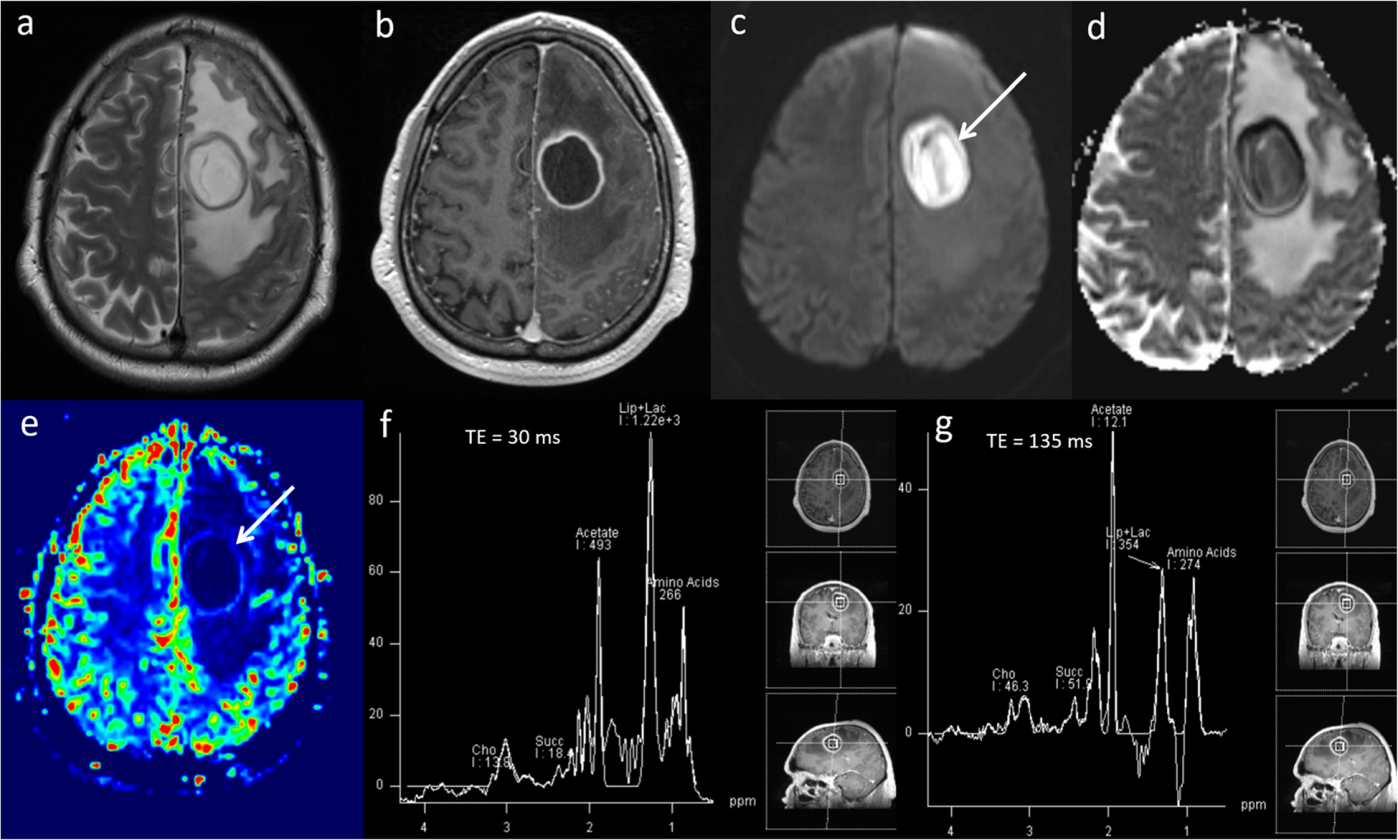
Abscess. Conventional MRI: **a**, **b** T2W and post-contrast T1W sequences demonstrate a ring-enhancing mass lesion in the left frontal lobe with surrounding oedema. **c**, **d** DWI and ADC sequences show low ADC (600 × 10^−6^ mm^2^ s^−1^, arrow) throughout the lesion. **e** PWI demonstrates significantly lower perfusion (arrow) than the contralateral white matter. **f**, **g** MRS shows high lipid as well as the presence of amino acid (0.9–1.0 ppm), acetate (1.92 ppm) and succinate peaks (2.42 ppm). These characteristic MRS findings in combination with the very low ADC and low perfusion are diagnostic of abscess. Diagnosis was confirmed on aspiration which revealed colonies of gram-positive cocci

Cerebral abscesses account for 1–8% of intracranial mass lesions [40]. Diagnosis can be challenging as abscesses on conventional imaging can mimic primary necrotic tumours and metastases. By using MRS and DWI, the sensitivity/specificity for diagnosis is up to 100% [41, 42]. Multiparametric MRI features of abscess are uniformly low ADC due to the higher viscosity of fluid. The ADC values are typically less than 700 × 10^−6^ mm^2^ s^−1^ [43], which is lower than expected to be seen in high-grade tumours or metastases (700–780 × 10^−6^ mm^2^ s^−1^). Perfusion at the margins and centre of the lesion is usually low. MRS features of abscess are different from tumours and show predominantly protein breakdown products on the right side of the ppm scale, including amino acid, acetate and succinate peaks as well as the presence of a lactate peak. Typical multiparametric appearances of an abscess are shown in Fig. 12.

### Tuberculoma

**Fig. 13:**
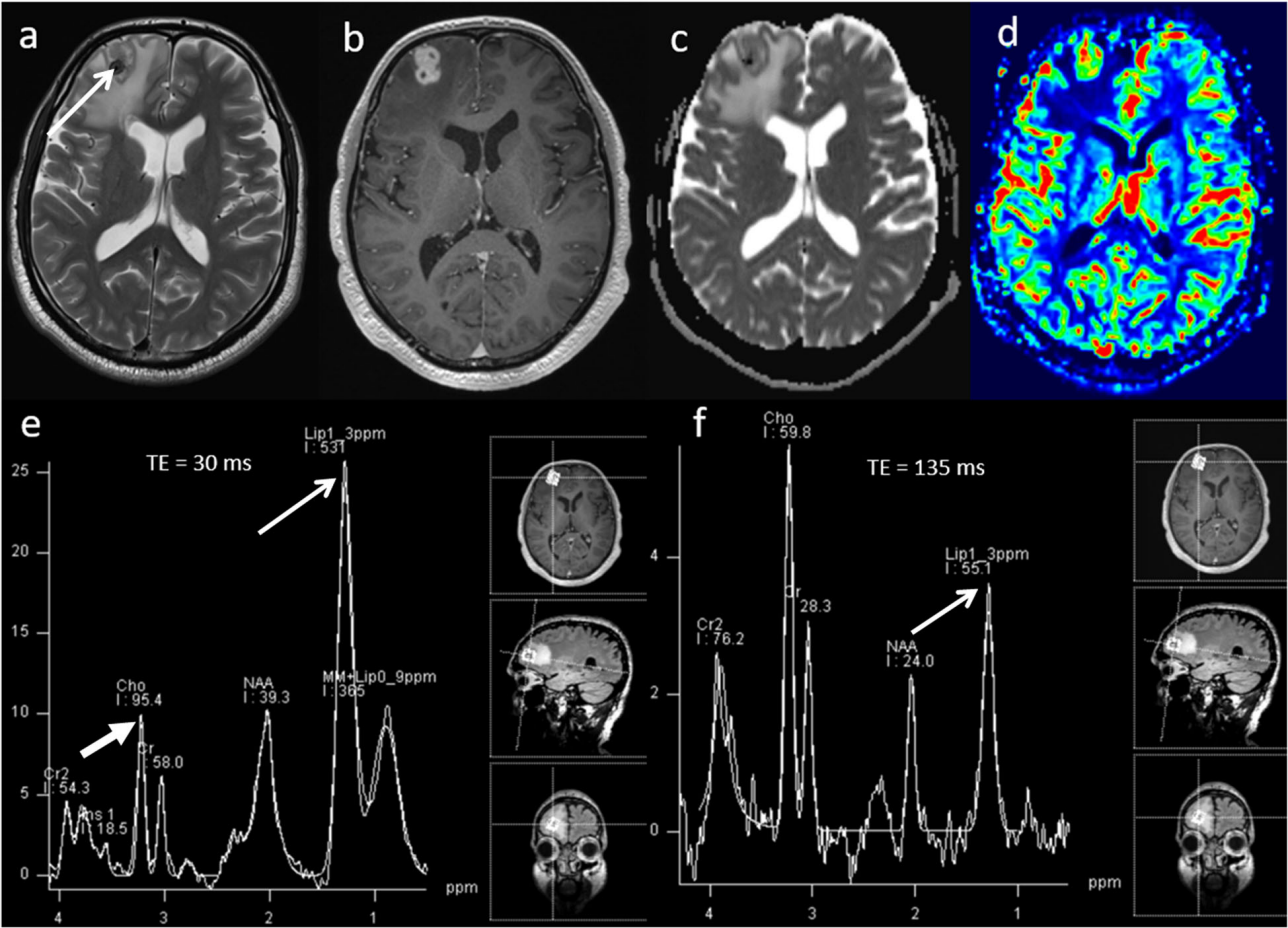
Tuberculoma. Conventional MRI Findings: **a**, **b** Axial T2W and post-contrast T1W sequences show T2W hypointense confluent lesions in the right frontal lobe with extensive perilesional oedema and enhancement. Multiparametric MRI: **c** ADC map shows intermediate values (900 × 10^−6^ mm^2^ s^−1^), (**d**) PWI shows perfusion higher than the contralateral white matter, (**e**, **f**) MRS shows very high levels of lipid at 1.3 ppm (thin arrows), without any lactate. There is slightly elevated Cho/Cr ratio (1.5) on short TE MRS (thick arrow), moderately low NAA/Cr ratio and absence of mI. In this case of tuberculoma, the combination of a T2W hypointense lesion, raised rCBV, raised lipids and moderately increased Cho/Cr ratio helped to make the diagnosis. The patient commenced anti-tuberculosis treatment, and surgical intervention was avoided.

Intracranial tuberculoma is a rare cause of a space-occupying lesion composed of caseating granuloma from systemic spread of tuberculosis infection, but potentially lethal as it can rupture and cause tuberculous meningitis. Conventional MRI appearances of tuberculoma include a T2W hypointense lesion with rim and ring enhancement. The differential diagnoses with this appearance are between tumour, abscess or granuloma. Multiparametric MRI usually demonstrates an intermediate level of ADC, elevated perfusion and high lipids on MRS, with a normal spectroscopic pattern in the perilesional area. ADC can be variable according to the stage of disease, degree of cellular infiltration and liquefactive necrosis [44]. Elevated rCBV is seen in tuberculoma, secondary to angiogenesis and inflammation. The lipids at 1.3 ppm seen on MRS in tuberculoma reflect the mycobacterium wall and moderately high choline is present due to inflammatory activity [45]. High-grade tumour has shown to demonstrate a higher mean Cho/Cr ratio compared to tuberculoma, 2.1 and 1.3 respectively on short TE MRS [46]. A case of tuberculoma is shown in Fig. 13.

### Neurosarcoidosis

**Fig. 14:**
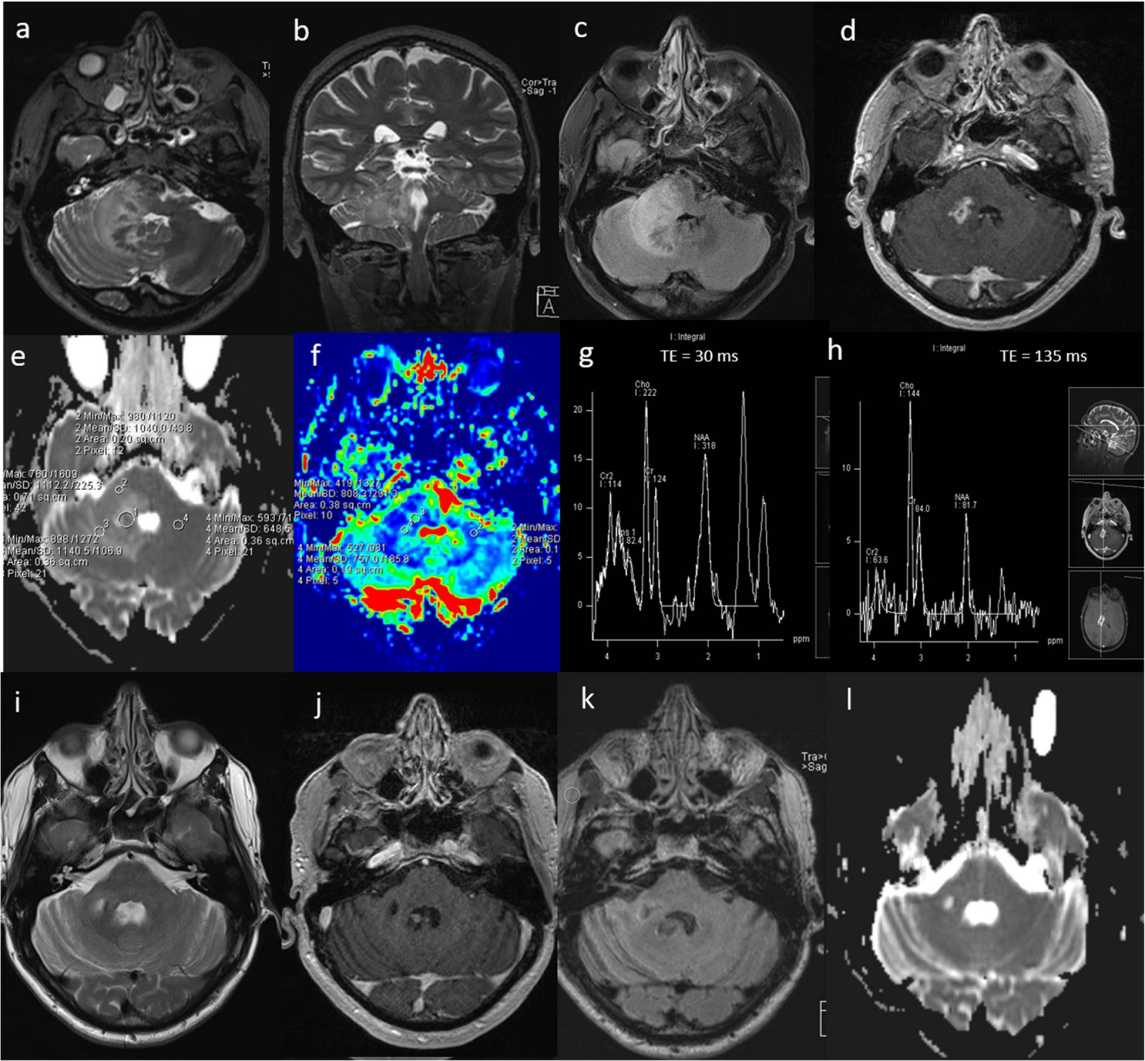
Neurosarcoidosis. Known case of systemic sarcoidosis. Conventional MRI Findings: **a**, **b** Axial and coronal T2W, (**c**) axial FLAIR and (**d**) post-contrast T1W sequences, showing a diffuse infiltrative lesion with enhancing foci in the right cerebellar peduncle extending to the brainstem, mimicking tumour. Multiparametric MRI: **e** DWI shows focal areas of low ADC. **f** PWI shows low perfusion in comparison to the contralateral side. **g** MRS with a short TE (30 ms) shows moderately high Cho/Cr ratio (< 2), near normal NAA/Cr and mI/Cr, presence of glutamate and glutamine at 2.4–2.6 ppm and large lipid peaks at 0.9 and 1.3 ppm suggesting necrosis. **h** MRS with a TE 135 ms shows slightly low NAA/creatine ratio and absence of lactate. In this case, the findings of low perfusion (< 2), absence of a lactate peak and presence of glutamine and glutamate favour an inflammatory aetiology such as neurosarcoidosis rather than a high-grade glioma. A tapering dose of oral prednisolone was commenced, during which neurological symptoms improved. Three-month follow-up MRI; (**i**) axial T2W, (**j**) post-contrast T1W, (**k**) FLAIR and (**l**) ADC sequences show near complete resolution of the lesion after treatment with steroids

Sarcoidosis is an idiopathic systemic disease with non-caeseating granuloma. It typically presents as multiple enhancing parenchymal and/or meningeal lesions and can be extremely difficult to differentiate from high-grade glioma and metastases. In our experience, multiparametric MRI usually shows focal areas of low ADC, low perfusion, moderately high Cho/Cr ratio, presence of glutamate and glutamine peak at 2.4–2.6 ppm, large lipid peaks at 0.9 and 1.3 ppm with an absence of a lactate peak suggesting necrosis. A case of neurosarcoidosis is demonstrated in Fig. 14a–h. Follow-up MRI shows near complete resolution of the lesion (Fig. 14i–l). A response to steroid treatment is usually helpful in making diagnosis.

### Encephalitis

**Fig. 15:**
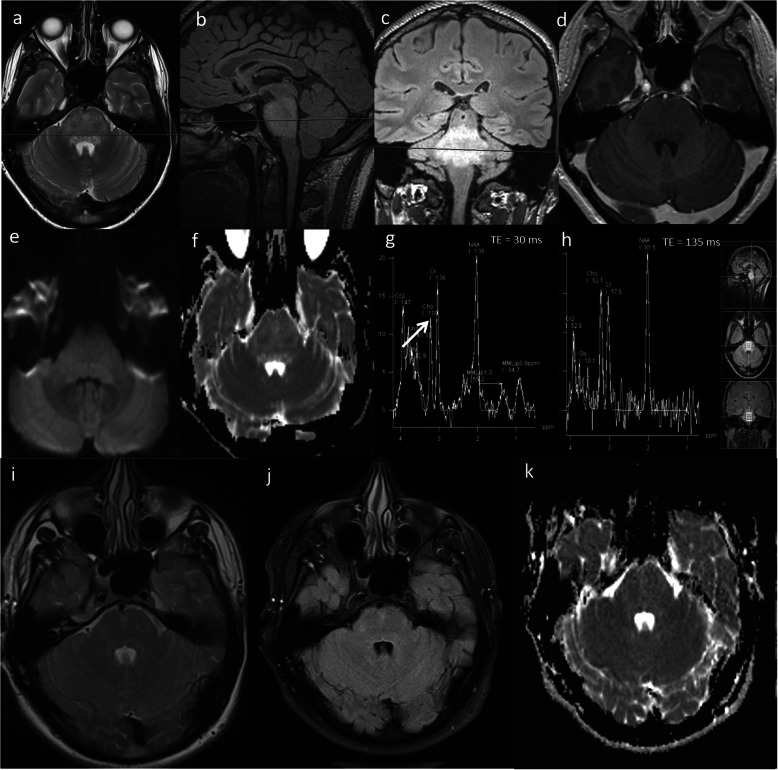
Bickerstaff brainstem encephalitis. Conventional MRI Findings: (**a**) Axial T2W, (**b**, **c**) sagittal and coronal FLAIR and (**d**) axial post-contrast T1W sequences, show a diffuse high signal lesion in the pons with no enhancement post-contrast. Multiparametric MRI: **e**, **f** DWI shows high ADC throughout the lesion (> 1000 × 10^−6^ mm^2^ s^−1^). **g**, **h** MRS shows normal mI/Cr, normal Cho/Cr (arrow) and normal NAA/Cr ratios and minimally increased glutamine and glutamate peaks (2.3 and 2.4 ppm). PWI (not shown) had low rCBV compared to normal-appearing white matter. The lack of enhancement, low rCBV, high ADC and normal choline exclude glioma. These multiparametric MRI features in conjunction with an acute presentation favour an inflammatory lesion. Two-month follow-up imaging: (**i**) axial T2W, (**j**) FLAIR and (**k**) ADC sequences show lesion regression and normalisation of diffusion. In this case, CSF analysis revealed antiganglioside antibodies consistent with a diagnosis of Bickerstaff brainstem encephalitis

Bickerstaff’s Brainstem encephalitis is a rare disorder characterised by acute ophthalmoplegia, ataxia and altered sensorium [47]. It is now increasingly being recognised as anti-GQ1b syndrome or spectrum disorder [48]. Brainstem signal abnormality has a wide differential of imaging appearances on conventional MRI and may mimic glial tumour. The treatment options of these entities vary significantly. A case of Bickerstaff brainstem encephalitis is shown in Fig. 15a–h*.* In this case, the lack of enhancement, low rCBV, high ADC, normal choline as well as presence of glutamine and glutamate at 2.3 and 2.4 ppm excluded glioma. Following treatment with intravenous methylprednisolone, follow-up MRI shows complete resolution (Fig. 15i–k).

### Tumefactive demyelination

**Fig. 16:**
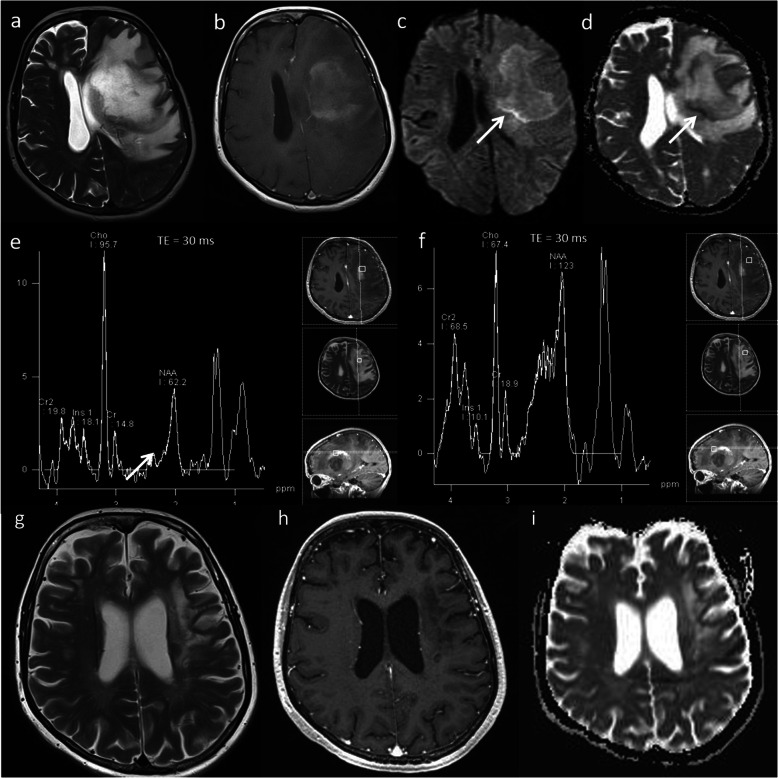
Tumefactive demyelination. Conventional MRI: **a**, **b** T2W and post-contrast T1W sequences reveals a large heterogeneous space occupying mass lesion and diffuse pattern of enhancement. Multiparametric MRI: **c**, **d** DWI and ADC images show high ADC centrally (> 1000 × 10^−6^ mm^2^ s^−1^) and a thin rim of low ADC reflecting advancing edge of demyelination (arrow). **e** MRS shows a high Cho/Cr ratio (6.4), near normal NAA/Cr ratio, high glutamate and glutamine (arrow), low mI/Cr ratio and the presence of lipid and lactate at 0.9 ppm and 1.3 ppm respectively. **f** The metabolic profile from the adjacent perilesional area also shows a similarly abnormal spectral pattern. PWI (not shown) demonstrated a low rCBV except in the anterior-superior component. The striking presence of glutamine and glutamate on MRS, the enhancement pattern and generally low perfusion favour an inflammatory lesion, as opposed to high-grade glioma or lymphoma. The patient made a recovery on methylprednisolone. One-month follow-up imaging: (**g**) Axial T2W, (**h**) post-contrast T1W and (**i**) ADC map shows significant improvement in mass effect, midline shift and overall volume of the lesion

Multiple sclerosis is a chronic inflammatory disease of the central nervous system. ‘Tumefactive demyelination’ is the term given when clinical and imaging findings are indistinguishable from those of a neoplastic mass lesion. This is estimated to occur in about 1–2 out of every 1000 cases of multiple sclerosis [49]. Acute tumefactive lesions can have ill-defined borders, mass effect, surrounding oedema, central necrosis and contrast enhancement, which mimic tumour [50]. They usually demonstrate central high ADC, a thin rim of low ADC (representing the active zone of demyelination), generally low rCBV, high Cho/Cr ratio, high glutamate and glutamine (demonstrating inflammatory activity) and presence of lipid and lactate. The metabolic profile from the adjacent perilesional area usually shows a similarly abnormal spectral pattern. MRS should not be read in isolation as it can mimic tumoural spectrum; however, the combination of parameters will lead to the correct diagnosis of tumefactive demyelination. A case of tumefactive demyelination is shown in Fig. 16a–f*.* The patient avoided biopsy and follow-up imaging shows significant improvement (Fig. 16g–i).

### Corpus callosum—epidermoid-like lesion

**Fig. 17:**
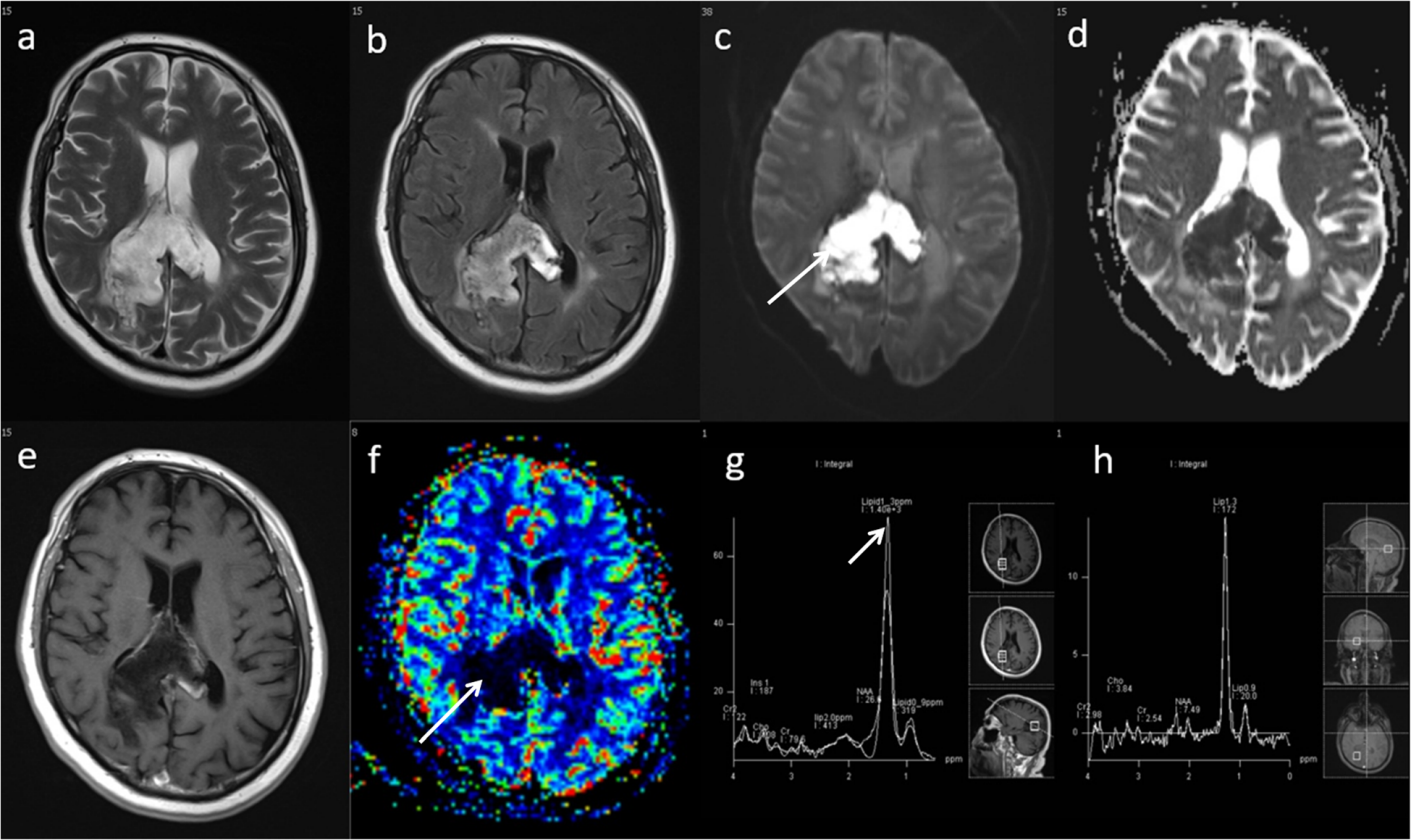
Epidermoid-like lesion of the corpus callosum. Conventional MRI: **a**, **b**, **e** T2W, FLAIR and post-contrast T1W sequences show a lesion involving the splenium of the corpus callosum and right parietal lobe. Multiparametric MRI: **c**, **d** DWI and ADC images show restricted diffusion (arrow). **f** Very low perfusion on PWI (arrow). **g** MRS shows very high lipid (1.3 ppm, arrow), without an increase in choline. In this case, appearances are not typical for high-grade glioma as there is low perfusion and no significant increase in choline, and it is not typical for lymphoma as there is no contrast enhancement or raised choline. Biopsy was consistent with epidermoid-like lesion with no evidence of tumour

The main differential diagnosis for a mass lesion involving the corpus callosum lesion is between glioblastoma and lymphoma. On conventional imaging, it is sometimes difficult to differentiate between these two entities and other less common lesions. Multiparametric MRI provides additional information to help in distinguishing benign from malignant lesions of the corpus callosum and tumoural from non-tumoural lesions. A case of a benign epidermoid-like lesion of the corpus callosum is shown in Fig. 17.

## Opportunities and challenges

There are some inherent challenges for adoption of multiparametric techniques in routine clinical practice, such as brain regions affected by susceptibility, small lesions and non-enhancing lesions. However, the adoption and widespread clinical use of multiparametric MRI protocols is improving with the use of higher magnetic field strength magnets, specialised coils and readily available vendor post-processing tools. We have incorporated a multiparametric MRI protocol consisting of DWI, PWI and MRS into our routine clinical practice for neuroimaging and our single-centre experience shows that these techniques clearly make a positive difference for individual patient management. It helps make more informed decisions at the tumour board (multi-disciplinary team) meetings, removing some uncertainty and leading to patients starting appropriate treatment earlier, which improves the overall survival rate and outcome. It is imperative that multiparametric information is read in combination with structural MR sequences, such as T1W, T2W, FLAIR, SWI, GRE to further characterise lesions. These semi-quantitative multiparametric parameters (ADC, rCBV, Cho) should be evaluated comprehensively and in conjunction with each other, rather than in isolation to narrow the differential diagnosis. With advances in these techniques, neuroradiology is in a unique position to evaluate the whole tumour and peri-tumoural environment, which could be a big limitation for histopathology, as commonly noticed in biopsy sampling error [26]. It is not uncommon for histopathology results to be re-reviewed following incorporation of these adjunct techniques in clinical practice, leading to a change in patient management.

There has been improvement in the standardisation of acquisition techniques over time, particularly with the publication of white papers on imaging [51, 52]. However, the cross-site and cross-vendor standardisation is still difficult to address, as there is some variability of threshold values and limited understanding of combining the parametric information. However, this will further improve with routine incorporation of these techniques in clinical practice with larger datasets and multi-centre studies.

## Conclusion

Through this educational pictorial review, we have presented a variety of cases to demonstrate that multiparametric MRI using DWI, PWI and MRS in conjunction with conventional MRI is helpful for differentiating neoplastic from non-neoplastic lesions in the brain. It also helps in the grading of tumours, selecting biopsy targets particularly in non-enhancing lesions and assessing treatment response. We have also presented a practical approach to perform multiparametric MRI protocol in routine clinical practice.

## Abbreviations

ADC: Apparent diffusion coefficient
AIF: Arterial input function
Cho: Choline
Cr: Creatine
DSC: Dynamic susceptibility contrast
DWI: Diffusion-weighted imaging
FLAIR: Fluid-attenuation inversion recovery
GE-EPI: Gradient-echo echo-planar imaging
Glx: Glutamine + glutamate
Gly: Glycine
GRE: Gradient echo
mI: Myo-inositol
MPRAGE: Magnetisation-prepared rapid acquisition with gradient echo
MRI: Magnetic resonance imaging
MRS: Magnetic resonance spectroscopy
MRSI: Magnetic resonance spectroscopic imaging
NAA: N-acetylaspartate
PCNSL: Primary central nervous system lymphoma
PRESS: Point resolved spectroscopy
PWI: Perfusion-weighted imaging
rCBF: Relative cerebral blood flow
rCBV: Relative cerebral blood volume
ROI: Region-of-interest
SRS: Stereotactic radiosurgery
SWI: Susceptibility-weighted imaging
T1W: T1-weighted
T2W: T2-weighted
TE: Echo time
WHO: World Health Organisation

## References

[1] Hall WA (1998). The safety and efficacy of stereotactic biopsy for intracranial lesions. Cancer.

[2] Malhotra HS, Jain KK, Agarwal A (2009). Characterization of tumefactive demyelinating lesions using MR imaging and in-vivo proton MR spectroscopy. Mult Scler.

[3] Hourani R, Brant LJ, Rizk T, Weingart JD, Barker PB, Horská A (2008). Can proton MR spectroscopic and perfusion imaging differentiate between neoplastic and nonneoplastic brain lesions in adults?. AJNR Am J Neuroradiol.

[4] Yang D, Korogi Y, Sugahara T (2002). Cerebral gliomas: prospective comparison of multivoxel 2D chemical-shift imaging proton MR spectroscopy, echoplanar perfusion and diffusion-weighted MRI. Neuroradiology.

[5] Di Costanzo A, Scarabino T, Trojsi F (2014). Recurrent glioblastoma multiforme versus radiation injury: a multiparametric 3-T MR approach. Radiol Med.

[6] Sawlani V, Davies N, Patel M (2019). Evaluation of response to stereotactic radiosurgery in brain metastases using multiparametric magnetic resonance imaging and a review of the literature. Clin Oncol (R Coll Radiol).

[7] Grech-Sollars M, Hales PW, Miyazaki K (2015). Multi-centre reproducibility of diffusion MRI parameters for clinical sequences in the brain. NMR Biomed.

[8] Kitis O, Altay H, Calli C, Yunten N, Akalin T, Yurtseven T (2005). Minimum apparent diffusion coefficients in the evaluation of brain tumors. Eur J Radiol.

[9] Abrigo JM, Fountain DM, Provenzale JM et al (2018) Magnetic resonance perfusion for differentiating low-grade from high-grade gliomas at first presentation. Cochrane Database Syst Rev. 10.1002/14651858.CD011551.pub2 10.1002/14651858.CD011551.pub2 PMC6491341 29357120

[10] Safriel Y, Pol-Rodriguez M, Novotny EJ, Rothman DL, Fulbright RK (2005). Reference values for long echo time MR spectroscopy in healthy adults. AJNR Am J Neuroradiol.

[11] Krukowski P, Podgórski P, Guziński M, Szewczyk P, Sąsiadek M (2010). Analysis of the brain proton magnetic resonance spectroscopy - differences between normal grey and white matter. Pol J Radiol.

[12] Usinskiene J, Ulyte A, Bjørnerud A (2016). Optimal differentiation of high- and low-grade glioma and metastasis: a meta-analysis of perfusion, diffusion, and spectroscopy metrics. Neuroradiology.

[13] Lee EJ, Lee SK, Agid R, Bae JM, Keller A, Terbrugge K (2008). Preoperative Grading of presumptive low-grade astrocytomas on MR imaging: diagnostic Value of minimum apparent diffusion coefficient. AJNR Am J Neuroradiol.

[14] Hakyemez B, Erdogan C, Ercan I, Ergin N, Uysal S, Atahan S (2005). High-grade and low-grade gliomas: differentiation by using perfusion MR imaging. Clin Radiol.

[15] Murakami R, Hirai T, Sugahara T (2009). Grading astrocytic tumors by using apparent diffusion coefficient parameters: superiority of a one-versus two-parameter pilot method 1. Radiology.

[16] Batchelor T, Loeffler JS (2006). Primary CNS Lymphoma. J Clin Oncol.

[17] Yamasaki F, Takayasu T, Nosaka R (2015). Magnetic resonance spectroscopy detection of high lipid levels in intraaxial tumors without central necrosis: a characteristic of malignant lymphoma. J Neurosurg..

[18] Saini J, Kumar Gupta P, Awasthi A (2018). Multiparametric imaging-based differentiation of lymphoma and glioblastoma: using T1-perfusion, diffusion, and susceptibility-weighted MRI. Clin Radiol.

[19] Pignatti F, van den Bent M, Curran D (2002). Prognostic Factors for Survival in Adult Patients With Cerebral Low-Grade Glioma. J Clin Oncol.

[20] Murphy ES, Leyrer CM, Parsons M (2018). Risk factors for malignant transformation of low-grade glioma. Int J Radiat Oncol Biol Phys.

[21] Rees J, Watt H, Jäger HR (2009). Volumes and growth rates of untreated adult low-grade gliomas indicate risk of early malignant transformation. Eur J Radiol.

[22] Bulik M, Jancalek R, Vanicek J, Skoch A, Mechl M (2013). Potential of MR spectroscopy for assessment of glioma grading. Clin Neurol Neurosurg.

[23] Scott JN, Brasher PM, Sevick RJ, Rewcastle NB, Forsyth PA (2002). How often are nonenhancing supratentorial gliomas malignant? A population study. Neurology..

[24] Danchaivijitr N, Waldman AD, Tozer DJ (2008). Low-grade gliomas: do changes in rCBV measurements at longitudinal perfusion-weighted MR Imaging predict malignant transformation?. Radiology.

[25] Soares DP, Law M (2009). Magnetic resonance spectroscopy of the brain: review of metabolites and clinical applications. Clin Radiol.

[26] Muragaki Y, Chernov M, Maruyama T (2008). Low-grade glioma on stereotactic biopsy: how often is the diagnosis accurate?. Minim Invasive Neurosurg.

[27] Jin T, Ren Y, Zhang H, Xie Q, Yao Z, Feng X (2019). Application of MRS- and ASL-guided navigation for biopsy of intracranial tumors. Acta Radiol.

[28] Georgakis MK, Spinos D, Pourtsidis A (2018). Incidence and survival of gliomatosis cerebri: a population-based cancer registration study. J Neurooncol.

[29] Förster A, Brehmer S, Seiz-Rosenhagen M (2019). Heterogeneity of glioblastoma with gliomatosis cerebri growth pattern on diffusion and perfusion MRI. J Neurooncol.

[30] Abbasi AW, Westerlaan HE, Holtman GA, Aden KM, van Laar PJ, van der Hoorn A (2018). Incidence of tumour progression and pseudoprogression in high-grade gliomas: a systematic review and meta-analysis. Clin Neuroradiol.

[31] Matsusue E, Fink JR, Rockhill JK, Ogawa T, Maravilla KR (2010). Distinction between glioma progression and post-radiation change by combined physiologic MR imaging. Neuroradiology.

[32] Hu LS, Baxter LC, Smith KA (2009). Relative cerebral blood volume values to differentiate high-grade glioma recurrence from posttreatment radiation effect: direct correlation between image-guided tissue histopathology and localized dynamic susceptibility-weighted contrast-enhanced perfusion MR imaging measurements. Am J Neuroradiol.

[33] Xu J-L, Shi D-P, Dou S, Li Y-L, Yan F (2011). Distinction between postoperative recurrent glioma and delayed radiation injury using MR perfusion weighted imaging. J Med Imaging Radiat Oncol.

[34] Sawlani V, Taylor R, Rowley K, Redfern R, Martin J, Poptani H (2012). Magnetic resonance spectroscopy for differentiating pseudo-progression from true progression in GBM on concurrent chemoradiotherapy. Neuroradiol J.

[35] Zeng Q-S, Li C-F, Liu H, Zhen J-H, Feng D-C (2007). Distinction between Recurrent glioma and radiation injury using magnetic resonance spectroscopy in combination with diffusion-weighted imaging. Int J Radiat Oncol Biol Phys.

[36] Hein PA, Eskey CJ, Dunn JF, Hug EB (2004). Diffusion-weighted imaging in the follow-up of treated high-grade gliomas: tumor recurrence versus radiation injury. AJNR Am J Neuroradiol.

[37] Song YS, Choi SH, Park C-K (2013). True progression versus pseudoprogression in the treatment of glioblastomas: a comparison study of Normalized cerebral blood volume and apparent diffusion coefficient by histogram analysis. Korean J Radiol.

[38] O’Beirn M, Benghiat H, Meade S (2018). The expanding role of Radiosurgery for brain metastases. Medicines (Basel).

[39] Patel TR, McHugh BJ, Bi WL, Minja FJ, Knisely JPS, Chiang VL (2011). A comprehensive review of MR imaging changes following radiosurgery to 500 brain metastases. Am J Neuroradiol.

[40] Osenbach RK, Loftus CM (1992). Diagnosis and management of brain abscess. Neurosurg Clin N Am.

[41] Hsu S-H, Chou M-C, Ko C-W (2013). Proton MR spectroscopy in patients with pyogenic brain abscess: MR spectroscopic imaging versus single-voxel spectroscopy. Eur J Radiol.

[42] Xu X-X, Li B, Yang H-F (2014). Can diffusion-weighted imaging be used to differentiate brain abscess from other ring-enhancing brain lesions? A meta-analysis. Clin Radiol.

[43] Horvath-Rizea D, Surov A, Hoffmann K-T (2018). The value of whole lesion ADC histogram profiling to differentiate between morphologically indistinguishable ring enhancing lesions-comparison of glioblastomas and brain abscesses. Oncotarget.

[44] Peng J, Ouyang Y, Fang W-D (2012). Differentiation of intracranial tuberculomas and high grade gliomas using proton MR spectroscopy and diffusion MR imaging. Eur J Radiol.

[45] Gupta RK, Roy R, Dev R (1996). Finger printing of mycobacterium tuberculosis in patients with intracranial tuberculomas by usingin vivo,ex vivo, andin vitro magnetic resonance spectroscopy. Magn Reson Med.

[46] Morales H, Alfaro D, Martinot C, Fayed N, Gaskill-Shipley M (2015) MR spectroscopy of intracranial tuberculomas: a singlet peak at 3.8 ppm as potential marker to differentiate them from malignant tumors. Neuroradiol J 28(3):294–302 10.1177/1971400915592077 PMC4757282 26246099

[47] Odaka M, Yuki N, Yamada M (2003). Bickerstaff’s brainstem encephalitis: clinical features of 62 cases and a subgroup associated with Guillain–Barré syndrome. Brain.

[48] Ogawara K, Kuwabara S, Yuki N (2002). Fisher syndrome or Bickerstaff brainstem encephalitis? Anti-GQ1b IgG antibody syndrome involving both the peripheral and central nervous systems. Muscle Nerve.

[49] Lucchinetti CF, Gavrilova RH, Metz I (2008). Clinical and radiographic spectrum of pathologically confirmed tumefactive multiple sclerosis. Brain.

[50] Saindane AM, Cha S, Law M, Xue X, Knopp EA, Zagzag D (2002). Proton MR spectroscopy of tumefactive demyelinating lesions. Am J Neuroradiol.

[51] Öz G, Alger JR, Barker PB (2014). Clinical Proton MR Spectroscopy in Central Nervous System Disorders. Radiology.

[52] Welker K, Boxerman J, Kalnin A (2015). ASFNR recommendations for clinical performance of MR dynamic susceptibility contrast perfusion imaging of the brain. AJNR Am J Neuroradiol..

